# Human age and skin physiology shape diversity and abundance of Archaea on skin

**DOI:** 10.1038/s41598-017-04197-4

**Published:** 2017-06-22

**Authors:** Christine Moissl-Eichinger, Alexander J. Probst, Giovanni Birarda, Anna Auerbach, Kaisa Koskinen, Peter Wolf, Hoi-Ying N. Holman

**Affiliations:** 1 Medical University of Graz, Department of Internal Medicine, Auenbruggerplatz 15, 8036 Graz, Austria; 2 BioTechMed-Graz, Mozartgasse 12/II, 8010 Graz, Austria; 3 Department of Earth and Planetary Science, University of California, Berkeley, 307 McCone Hall, Berkeley, CA 94720 USA; 4 Elettra – Sincrotrone Trieste, Strada Statale 14 - km 163,5 in AREA Science Park, 34149 Basovizza, Trieste Italy; 5 University of Regensburg, Department of Microbiology and Archaea Center, Universitaetsstr. 31, 93053 Regensburg, Germany; 6 Medical University of Graz, Department of Dermatology, Auenbruggerplatz 8, 8036 Graz, Austria; 7 Berkeley Synchrotron Infrared Structural Biology Program, Lawrence Berkeley National Laboratory, One Cyclotron Road, Berkeley, California United States of America

## Abstract

The human skin microbiome acts as an important barrier protecting our body from pathogens and other environmental influences. Recent investigations have provided evidence that Archaea are a constant but highly variable component of the human skin microbiome, yet factors that determine their abundance changes are unknown. Here, we tested the hypothesis that the abundance of archaea on human skin is influenced by human age and skin physiology by quantitative PCR of 51 different skin samples taken from human subjects of various age. Our results reveal that archaea are more abundant in human subjects either older than 60 years or younger than 12 years as compared to middle-aged human subjects. These results, together with results obtained from spectroscopy analysis, allowed us gain first insights into a potential link of lower sebum levels and lipid content and thus reduced skin moisture with an increase in archaeal signatures. Amplicon sequencing of selected samples revealed the prevalence of specific eury- and mainly thaumarchaeal taxa, represented by a core archaeome of the human skin.

## Introduction

The human skin is our primary interface to the external environment. With a total area of 1.8 m^2^ and the abundance of folds, invaginations and appendages, the skin is populated by a diverse community of microorganisms termed the skin microbiome. The overwhelming majority of these microorganisms are commensals, which impede the invasion of more pathogenic species, enable host surface-microbe interactions, and provide vital functions to the overall cutaneous health^1^.

The skin microbiome composition depends largely on cutaneous structure and chemical composition, reflected by the location on the body, skin constitution (epidermis or dermis), appendages (glands or follicles), and skin topographical variability (e.g. moist or dry sites)^1–5^. For example, bacterial populations differ between sebaceous (oily, waxy), moist or dry skin areas. *Propionibacterium* and *Staphylococcus* were reported to be found mainly in sebaceous skin sites such as the face and torso, whereas *Corynebacterium*, *Staphylococcus* and different ß-Proteobacteria in moist areas (armpit, arm and knee fossa). Dry areas also often have the richest microbial communities^6^ and seem to be dominated by β-Proteobacteria, *Corynebacterium* and Flavobacteriales^1^.

The skin microbiome composition is also influenced by host factors including immune system, age and sex^6^. The skin of a new-born first adopts the microbiome of either mother’s vagina, or the skin microbiome of family members in case of a Caesarean section^7^. The baby’s microbiome changes rapidly within the first months, develops further in the following years and reaches an adult microbiome composition at sexual maturity^7, 8^.

The effect of sex on the human microbiome was found to substantially influence the microbial community composition of skin, most likely due to different steroid productions^6, 9^. This sex effect is not limited to the microbial palm community^4^, it exists almost in all body sites, especially the glabella area^2^, where the cosmetics application could be a trigger^10^. The influence of age and sex on the skin microbiome composition was further confirmed in a recent study which took living locations into account^2^. By comparing 71 human subjects from urban and rural regions of Shanghai in China, a significantly more diverse microbial community was found on the skin of adult humans (25–35 years old) relative to adolescents (12–19 years old) and elderly (50–60 years old), which was explained by the acclimation of the bacterial communities to changes with age in skin conditions such as skin moisture or sebum levels.

The human skin microbiome consists of Bacteria, Archaea, Eukaryotes and viruses. Currently, skin microbiome studies focus largely on bacterial constituents and recently on viruses perturbation to the skin bacteriome^11^, and less on skin Eukaryotes such as fungi^8, 12^. Archaea, forming a separate domain of life, are even less considered.

So far, only a few studies reported the occasional detection of archaea on human skin^13–15^: Thaumarchaeota have been detected in a study of palms of two individuals^14^, whereas three different phylotypes of Archaea (two methanogens and one halophilic archaeon) appeared marginally in a large subset of bacterial sequences obtained from samples of 60 navels^13^. Archaea, appear mostly underestimated with respect to their abundance and functional role, even though the human body harbours a range of different archaea as it has been shown for the gut, mouth and vaginal microbiome. In particular, methanogenic archaea (genera *Methanobrevibacter*, *Methanosphaera*, *Methanosarcina*) seem to be the major archaeal representatives.

It is generally assumed, that Archaea are a minor, transient part of the human associated skin microbiome playing an insignificant role. In the two studies mentioned above^13, 14^, Archaea were detected by means of co-amplification of their 16S rRNA genes along with Bacteria. In general, these types of protocols currently used for the analysis of the human microbiome are biased towards the detection of Bacteria – and consequently overlook the presence of Archaea due to, e.g., DNA extraction procedures that are often unsuitable for archaeal cell lysis, or due to primer mismatches. For instance, the primer pair F515/R806 that is generally used to amplify 16S rRNA genes from human microbiome samples, hits only 50% of all known Archaea sequences and only 8% of all Thaumarchaeota without mismatch^15^. These issues, in addition to the difficulties in cultivating archaea and microorganisms from skin, have led to an underestimation of the presence of skin archaea and their role in human skin health and disease is still a mystery.

First indications of a possibly larger role of skin-associated archaea arose from studying the microbiome in well-controlled cleanroom facilities that are predominantly influenced by human skin microbiota^16, 17^. Since two intensive care units in hospitals and different space exploration-related cleanrooms revealed the presence of Archaea, we suggested an association of Thaumarchaeota with humans, and most likely with human skin^15^. This assumption was proven by analyzing skin samples from the torsos of seven female and six male human subjects, who were all found to be positive for Archaea^15^. On some individuals we identified the relative abundance of archaeal 16S rRNA genes to be 4.4% compared to the total recovered prokaryotic 16S rRNA genes; on average, each human sample showed 0.7% archaeal gene signatures (corresponding to approx. 1.7% of all cells^15^). The archaeal diversity analyzed in five selected human skin samples revealed lineages of the phyla Euryarchaeota (12% of all lineages, *Methanosarcina*) and Thaumarchaeota (88% of all lineages). Detailed phylogenetic analyses of the archaeal skin sequences placed the latter within the so-called group I.1b (“*Nitrososphaera* cluster”^18^; former “soil crenarchaeotic group”, SCG) of Thaumarchaeota, close to sequences previously found in artificial environments and soils^15, 19^. The presence of skin archaea was further confirmed by fluorescence *in situ* hybridization (FISH) which revealed small coccoid-shaped cells that are morphologically in high accordance with the description of *Nitrososphaera viennensis* ^15, 20^. The great variation of archaeal abundances across previous samples^15^ and the general dependency on age of the skin microbiome^2^ motivated the current study.

Here, we report our investigation of Archaea in skin microbiome samples from torsos of 51 volunteers in the age of 1 to 75 years. Beyond DNA-based approaches that included quantitative PCR and next generation sequencing of 16S rRNA genes, we also used Fourier Transform infrared (FTIR) focal plan array (FPA) hyperspectral imaging to detect Archaea in human skin samples. The unique infrared spectral signature of archaeal membrane lipids is used to detect their presence in environmental samples^21, 22^. Overall, we present molecular and spectroscopic evidence that skin physiology and age possibly shape the diversity and abundance of Archaea on skin.

## Results

### Archaeal abundance is dependent on the age of human subjects

**Figure 1:**
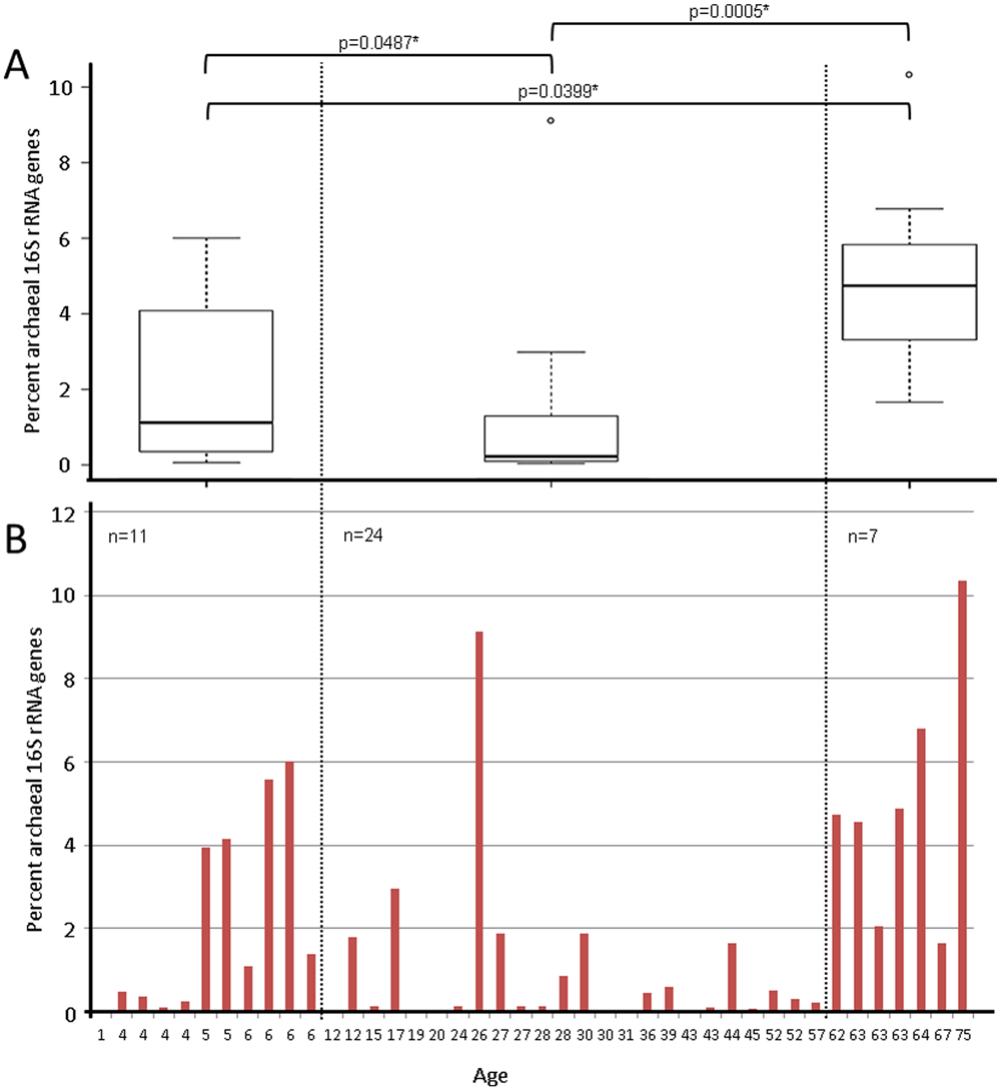
(**A**) Box-Whisker plot of fraction of archaeal 16S rRNA genes (as percentage of total 16S rRNA genes detected by quantitative PCR) detected in skin wipe samples taken from volunteers of different age (in years). The differences between the three age-groups (indicated by vertical lines) were statistically significant. Outliers are shown as circles. (**B**) Proportion of archaeal 16S rRNA genes amongst all detectable microbial signatures in each of the samples. X-axis gives the actual age of the volunteer.

In order to investigate the hypothesis that the archaeome is dependent on the age of the human subject, we retrieved quantitative measures on the archaeal and bacterial abundance in torso skin samples from qPCR. Solid qPCR data were retrieved from 42 out of 51 collected skin-wipe samples and were thus processed further (Overview of all samples given in Supplementary Table S1, Results of qPCR given in Supplementary Table S2). The samples showed a high variation in total 16S RNA gene copy numbers, ranging from 7.3 × 10^4^ to 1.1 × 10^8^ copy numbers per sample (Supplementary Table S2, Fig. 1). The relative abundance of archaeal 16S rRNA gene copies was found to be between”below detection limit” and 10.4%.

The results from Archaea-targeted quantitative PCR revealed no statistical difference between female and male volunteers. However, significant differences were found between age-groups, as presented in Fig. 1. The median of the archaeal fraction with respect to total microbial 16S rRNA gene copy numbers was 1.1% for the age group of 1–11 years (I), 0.2% for the age group 12–60 (II), and 4.7% for the 61 to 75 year old people (III). The differences between the three age-groups were statistically significant: p = 0.0487 (group I vs. II), p = 0.0399 (I vs. III) and p = 0.0005 (II vs. III). Thus we conclude that there exists causality between age and the percent Archaea on human skin.

### FTIR hyperspectral imaging allows a DNA-independent detection and chemistry understanding of Archaea in human skin-wipe samples

Beyond DNA-based approaches (quantitative PCR and next generation sequencing of 16S rRNA genes, see below), we also used Fourier Transform infrared (FTIR) focal plan array (FPA) hyperspectral imaging to: (1) confirm the presence of Archaea on human skin, and (2) provide an additional associative analysis of the conditions affecting the presence of the Archaea in the human skin microbiome. In general, the unique infrared spectral signature of archaeal membrane lipids can be used to detect their presence in environment samples of various origin (for detailed information see also Supplementary Fig. 1)^21, 22^.

The presence of Archaea on skins of the 10 independently sampled individuals (Supplementary Tables S1 and S3) was confirmed by FTIR-FPA hyperspectral measurements. In this study, we used this method as an imaging-based tool to detect the presence of Archaea (and Bacteria) in each binned pixel, and to identify chemical parameters associated with the cells. The ratio of the infrared absorbance in the −CH_3_ region (2,990–2,945 cm^−1^) to that of the −CH_2_ region (2,945–2,900 cm^−1^) was computed for each binned pixel (see materials and methods) and a value above 0.65 indicated the presence of Archaea therein.

**Figure 2:**
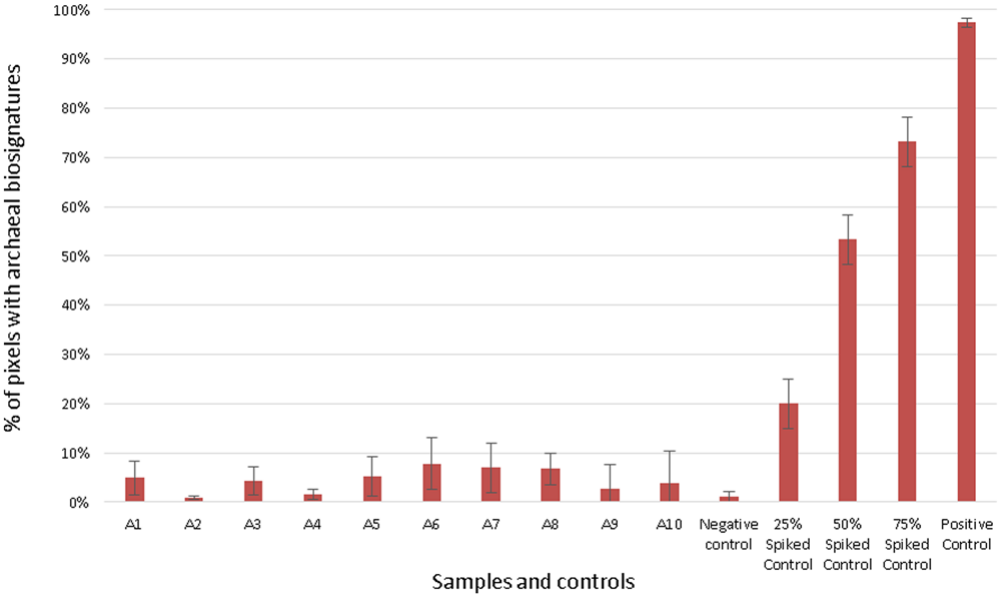
Count of pixels bearing archaeal signals, as retrieved by FTIR-FPA hyperspectral measurements, which was based on CH_3_/CH_2_ ratio of lipids. A1 to A10 are samples from different individuals. As negative control, sample buffer, without sample, was used. Due to unavoidable instrumental error and electrical noise, we anticipate a measurement error of about +/−1%. Positive controls were different concentrations of *Nitrososphaera viennensis* cells in buffer (25%, 50%, 75% and 100%).

Within this 10 individuals group, FTIR-FPA hyperspectral imaging measurements confirmed the presence of Archaea in all 10 samples with the relative abundance ranging from ∼1% to ∼8% of the total cells-containing pixels (see Fig. 2). This finding was consistent with the relative abundance of archaeal 16S rRNA gene copies compared to the entire microbial signatures (Fig. 1).

An additional analysis of ∼450.000 spectra revealed variability in the amount of lipids in our samples. From the Beer-Lambert Law, the integrated absorption intensity of lipids methyl (−CH_3_) and methylene (−CH_2_) groups in 3.000–2.800 cm^−1^ region is proportional to the amount of lipids in the sample. Lipids generally have the role of acting as a protective barrier^23, 24^. For example, the lipid-to-protein ratio was 1.03 for individual A4 (female, 64-year old) and 0.51 for A1 (male, 62-year old) although A1 had almost twice the biomass (as measured in absorption intensity; see Supplementary Table S4). It shall be noted, that subject A8 revealed the lowest value of lipids/proteins ratio, a mere 0.35. We interpreted that the observed low value of 0.35 is an indication that the A8 skin microenvironment was much drier than the others. This lowest value of the lipids/proteins ratio coincided with the highest number of archaeal 16S rRNA gene copies detected by quantitative PCR and high archaeal abundance as revealed via FTIR-FPA hyperspectral imaging (highlighted in Supplementary Table S4). Overall, this method indicated a possible trend that higher amounts of archaeal biosignatures are linked to dryer skin (lower lipid content; Supplementary Fig. S2).

### Thaumarchaeota are the major archaeal players on human skin

Representatively, we sequenced the archaeal 16S rRNA gene pool of 21 samples to investigate the phylogenetic pattern, and taxonomic distribution of Archaea across different ages. Overall, more than 770,000 reads were obtained via 454 sequencing from 21 samples (overview given in Supplementary Table S1), which were selected for NGS, with an average read length of 448 bp. However, one single sample (14.m.28) did not reveal any archaeal reads, and five out of 21 samples yielded a low number of archaeal reads only (<100; Supplementary Table S5), so that our analyses resulted in 15 solid archaeome datasets.

Bacterial amplicons were produced from samples 45.f.12 and 49.m.19 as amplification controls and confirmed previous reports on the bacterial skin microbiome as dominated by signatures from *Staphylococcus*, *Corynebacterium* and *Propionibacterium* (Supplementary Table S6). The extraction blank controls did not contain any bacterial sequences that were also found in the skin-wipe samples, and no archaeal reads were detected in the archaeal PCR negative control.

For further analysis of the archaeal sequences, only reads were considered, which appeared at least five times or more in the dataset. This resulted in an overall number of 196 archaeal OTUs (at 97% 16S rRNA gene sequence identity), with a total of almost 40,000 reads.

**Figure 3:**
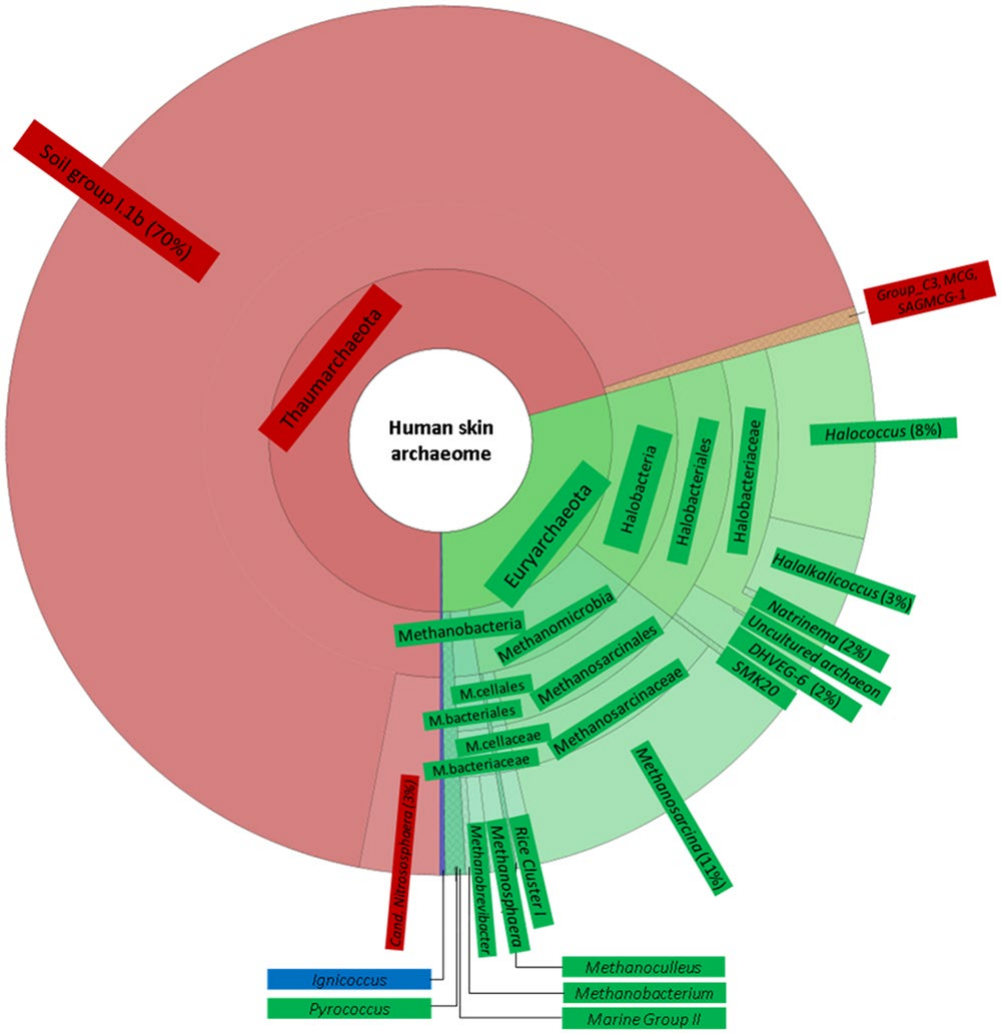
Hierarchical display (Krona^53^ diagram) of the human skin archaeome as revealed in this study. This chart is based on all archaeal sequences (reads) obtained via next generation sequencing of 15 skin wipe samples from volunteers in the age of 1 to 67 years. Thaumarchaeota are shown in red, Euryarchaeota in green, Crenarchaeota are indicated in blue.

**Figure 4:**
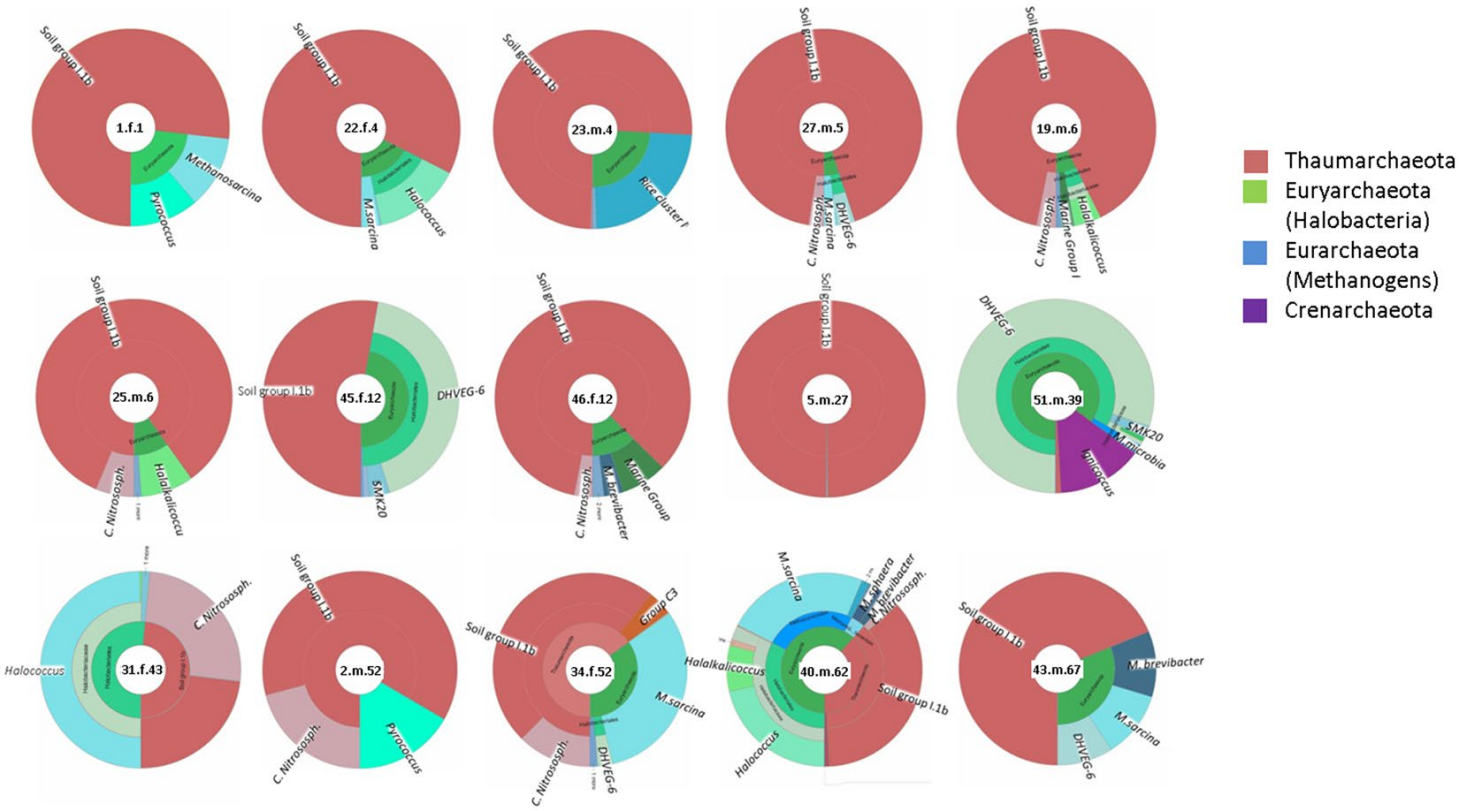
Hierarchical display (Krona^53^ diagrams) of the human skin archaeomes of different skin wipe samples. This chart is based on archaeal sequences (reads) obtained via next generation sequencing of 15 skin wipe samples from volunteers in the age of 1 to 67 years. Sample names, given in the middle of each chart, refer to sample number, sex (f = female, m = male), and age (separated by “.”).

A taxonomic summary of the discovered human skin archaeome is given in Fig. 3 (summary of all reads obtained from the 15 solid datasets), Fig. 4 (the 15 samples are displayed separately) and Supplementary Fig. S3 and S4 (bar chart/bubble plot of all reads obtained from all archaea-positive samples). Overall, 63 reads were attributed to Crenarchaeota, 11,689 reads belonged to Euryarchaeota and 28,246 were of thaumarchaeotal origin. The dominant and most abundant OTU with 3,770 total reads was detected in 15 of 20 samples (see Supplementary Table S5). This OTU was classified as representative of Thaumarchaeota from Soil Group I.1b, confirming the findings from our earlier study, which indicated that close relatives of *Nitrososphaera viennensis* are the most abundant archaea on human skin^15^. The highest number of archaeal OTUs retrieved from an individual sample was 123 (62 year old male, Supplementary Table S5).

Two people carried Crenarchaeota signatures on their skin (Supplementary Table S5, Fig. 4; 39 and 62 year old male volunteers), comprised in two OTUs classified as *Ignicoccus* (*Desulfurococcaceae*). Signatures of Euryarchaeota were much more abundant than Crenarchaeota. In particular, OTUs belonging to methanogenic archaea and Halobacteria were found in almost every sample. Methanogenic archaea (methanogens) are anaerobic, methane-producing microorganisms. Halobacteria, despite their confusing denotation, also belong to the archaeal phylum and contain halotolerant and halophilic species that thrive in salty ecological niches. Amongst the methanogens, mostly signatures from *Methanosarcina* species, as well as *Methanosphaera*, *Methanobrevibacter* and *Methanobacterium* were retrieved. Halobacteria were found in all samples, except in samples from the one year old and the 28 and 67 year old volunteers, with signatures from *Halococcus*, *Halalkalicoccus* being the most abundant in the datasets. Thaumarchaeota were found in each of the samples, except sample 16.w.28, whose archaeal skin microbiome was mainly composed of halophilic and methanogenic archaea. Most thaumarchaeal signatures retrieved from the other samples were classified as Soil Group I.1b Thaumarchaeota, while few signatures were also classified as MCG (miscellaneous crenarchaeotic group, now Bathyarchaeota), Group C3 or SAGMCG (South African gold mine archaea).

Calculation of the inverse Simpson and Shannon indices indicated a different diversity and richness profile for the three age groups (inverse Simpson: p = 0.0963 (anova), Shannon: p = 0.0348 (anova)), but no significant differences depending on sex (Supplementary Fig. S5 and S6). Overall, the archaeal diversity appeared to be lower in age group II (12–60 years), correlating with the low abundance of archaeal signatures in this age group as found by qPCR. A potential grouping of the age groups based on the archaeal community composition was confirmed by redundancy analysis (p = 0.028), as shown in Supplementary Fig. S7.

**Figure 5:**
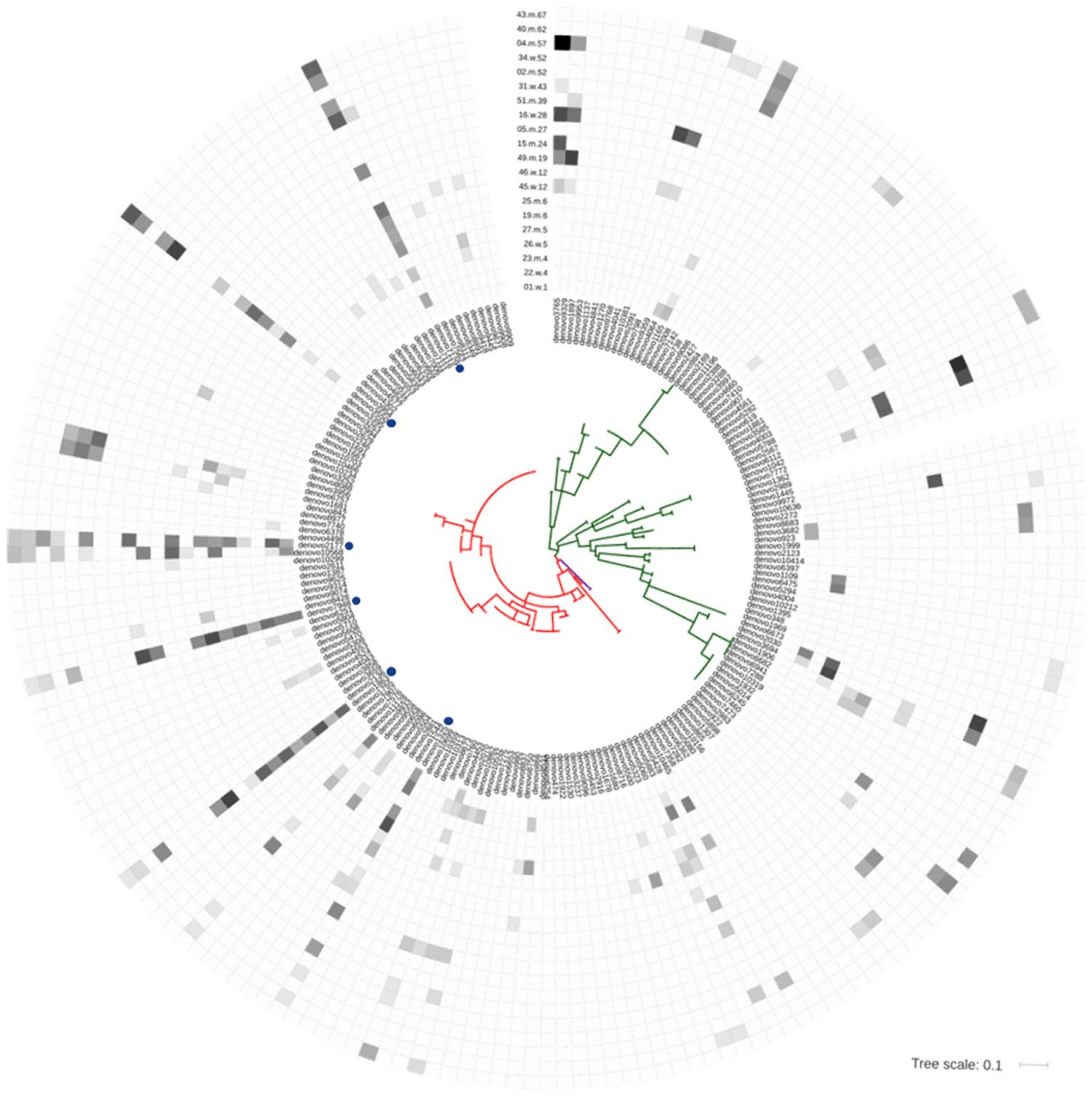
Phylogenetic tree of NGS-retrieved archaeal OTUs. The heatmap displayed on the edge of the tree reflects the percentage of reads retrieved from the respective OTU from each of the archaea-positive skin wipe samples (dark color indicates high abundance). Green branches: Euryarchaeota, red branches: Thaumarchaeota, purple branches: Crenarchaeota. Blue dots indicate OTUs that were found in at least 70% of all samples.

Overall and as shown in Fig. 5, some of the archaeal OTUs frequently appeared across different age groups. In particular, some thaumarchaeal sequences, such as *denovo*7129, *denovo*2177, *denovo*1863, *denovo*3550, *denovo*7948, *denovo*1325, were found in most of the samples, indicating that these OTUs are frequent or even general members of the human skin microbiome (core archaeome, threshold 70%; Fig. 5). The sequences clustered with environmental sequences (mostly from soil, also air filter samples) and with sequences obtained in our previous study^15^.

## Discussion

Based on our results presented above, we can state, that a thaumarchaeal core archaeome is part of the human skin microbial community through the entire life-span. Until now, the presence of Archaea on human skin has been largely overlooked in human microbiome research. Oh *et al*.^25^, for instance, reported the (near) absence of Archaea on the skin from healthy volunteers in the age of 23 to 39 years when metagenomic analysis was performed^25^.

The presence of Crenarchaeota-specific biosignatures in human samples have been reported rarely and for faeces samples only^26^. Some of the sequences retrieved in that study were found to be related to the genus *Sulfolobus*; other sequences obtained affiliated to Thaumarchaeota (I.1b) (former Crenarchaeota). In our study, the sequences from Crenarchaeota appeared only rarely, and their linkage to the human microbiome remains unclear. Within the detected Euryarchaeota, *Methanobacterium* has thus far not been reported for the human microbiome, whereas the other methanogenic genera have been detected previously in gut (all), mouth cave (*Methanosarcina*) and skin (also *Methanosarcina*)^27^. All described methanogens were characterized to be strict anaerobes (i.e. growth is absent when oxygen is present), and thus their presence on human skin appears to be transient. However, longitudinal studies and probably also cultivation or live-dead discrimination methods have to be applied in order to determine the physiological status of the methanogens on human skin. Other frequently found archaeal signatures from human skin samples were affiliated to haloarchaea. *Halococcus* signatures have already been reported to be present in human gut samples^28, 29^, and *Halalkalicoccus* has been placed in context with human skin microbiome due to the frequent appearance in cleanrooms that are populated by skin microorganisms^17^. Signatures of halophilic archaea associated with humans were first described in human faecal and mucosal samples^26, 28, 30, 31^. In particular, a study of stool samples from healthy Koreans revealed sequences related to *Halorubrum* and *Halococcus* ^30^. These sequences were also detectable in salt-fermented seafood, which was considered to be the source of these archaea or their DNA in the human stool samples^30^. Similar conclusions were drawn in a study, which found haloarchaea to be detectable in mucosa samples of patients that suffered from inflammatory bowel disease^28^. Possible sources like table salt or pre-endoscopic lavage solutions were discussed; however, the 16S rRNA gene sequences from mucosa and lavage solutions were not overlapping. Oxley *et al*.^28^ even managed to enrich *Halosimplex* sp. from a mucosal sample, proving that these organisms are persisting or even thriving in the human gut. In a very recent study, Khelaifia and Raoult reported the successful isolation of *Haloferax massiliensis* from a stool sample of an obese female patient. Despite the short report of having isolated this archaeon, no further information is currently available^29^. To date, the role of halophilic archaea in human samples remains unclear. The skin, however, is characterized by elevated salt concentrations from time to time (up to several percent; ref. 32), which could support the existence of such archaea in this environment, probably via microhabitats.

FTIR-FPA hyperspectral imaging was applied here as the third independent method following DNA-based analysis and fluorescence *in situ* hybridization^15^, which has confirmed the presence of archaeal signatures in samples from human skin. This non-invasive technique, due to its fast acquisition and analysis time and multiplex capability of providing chemical information, has proven to be an ideal screening tool. Here, this method indicated a possible trend that higher amounts of archaeal biosignatures are linked to dryer skin (lower lipid content; Supplementary Fig. S2), a situation that is often found associated with age.

Literature reports on age-dependent distribution of human-associated archaea are rare. Mihajlovski *et al*., have reported the appearance of Methanomassilicoccales to be significantly increased in elderly volunteers^33^. In the aforementioned study, the authors compared newborns (3 weeks to 10 months), adults (25–45 years) and elderly (70–90 years) with respect to the detectability of the *mcrA* gene in stool samples. Only 1 of 23 newborns revealed a positive signal, whereas 60% of the adults and 80% of the elderly subjects were positive for *mcrA* genes. This finding was in congruence with earlier reports on an increasing number of cultivable methanogens from rat gut samples with age^34^, or increased methane emission in breath of elderly^35^.

However, the analysis of 1,200 clones in the former study revealed, that the human-associated Methanobacteriales representatives (*M*. *smithii* and *M*. *stadtmanae*) were present without significant differences in adults and elderly. Nevertheless, representatives of the Methanomassiliicoccales were found in higher frequency in elderly people (40%, p = 0.065), but reasons for this increase remained undiscussed.

Several potential explanations may account for the triphasic distribution of Archaea on human skin with high levels of prevalence and diversity in childhood, low levels in adolescence and middle-aged subjects, and high levels in elderhood, entirely complementary to the diversity distribution of bacteria^2^. Variations in skin surface pH, sebum content and stratum corneum hydration have been reported with age, most likely related to endogenous steroid hormones^36–38^. Said so, in one study of an European population involving only females sebum production was highest in the age between 40 and 49 years, remained high till the age of 60 to 70 years, and significantly decreased thereafter^37^. Skin surface pH remained unchanged from 20 to 50 years, but increased significantly later in life^37^. In overall, similar age-related differences of sebum content, surface pH, and stratum corneum hydration of the skin were found in a study of large Chinese population comparing females and males^38^. Based on our results from FTIR-FPA hyperspectral imaging and quantitative PCR, one may speculate that a low sebum content and possibly also higher surface pH of the skin may favour the presence of Archaea. The hypothesis of involvement of the physiologic conditions of the skin in differential distribution of Archaea is consistent with the observation in a 26-year old female of our study as an outlier with high archaeal gene presence (Fig. 1), in whom post-hoc history revealed that she suffered from atopic dermatitis, a condition characterized by dryness with reduced sebum content and increased surface pH of the skin^39^.

The human skin bacteriome is highly individual and mainly dependent on specific host factors (hormone status, immune status, pathophysiology etc.), as well as body site, age, gender and place of residence^2, 40^. Although females and males have a different skin physiology in general^41^, and indications for sex-dependent differences in the skin bacteriome have been reported previously^4^, gender seemed not to have a significant impact on the archaeal diversity and community composition in our study. However, independently from gender, during puberty the sebum level increases, usually followed by an increase of lipophilic microorganisms on skin, such as *Propionibacterium* or *Malassezia* ^40^. In particular *Propionibacterium* has been reported to be able to hydrolyse triglycerides in human sebum and using those as a nutrient source^42, 43^. As archaea appear outside of the sebum/*Propionibacterium* phase, one might envisage an opposed or antagonistic behaviour, indicating a non-lipophilic life-style of the archaea.

So far, all cultivated group I.1b Thaumarchaeota have the capability of ammonia-oxidation. In detail, they perform nitrification, which means that they oxidize ammonia under aerobic conditions while producing nitrite. In general, they are autotrophs, but dependence on syntrophic interaction and/or the need for low amounts of organics has been reported^20, 44–46^. Thaumarchaeota have been generally described to thrive in natural environments, such as soil and aquatic environments. Their role on the human skin is yet to be determined and awaits further studies. However, due to their typical metabolism, one can also envisage ammonia oxidation on skin. This assumption is supported by the detection of *amoA* gene signatures in our previous study^15^. It shall be noted here, that the activity of nitrification, as proposed also for the skin Thaumarchaeota, actually goes along with a decrease in pH and thus could have a positive effect on the skin barrier. Sweat components, such as urea are considered possible nitrogen compounds for nitrification^20, 47^. One might envisage to apply skin-thaumarchaeal strains as skin-archaebiotics to potentially reduce body odor and decrease pH^48^, equivalent to the potential application of Methanomassiliicoccales for trimethyl-amine (TMA) removal in the gut^49^.

High pH, low stratum corneum hydration and reduced skin surface lipid content correlate with each other^37^ and may be crucial for the induction of antimicrobial peptides of the innate immune response^39^. In addition, earlier studies have indicated that Langerhans cells in the skin^50^ and the acquired immune response as measured by contact hypersensitivity may decline with age^51^ what may also play a role in the growth of the microbiome of the skin. Other potential explanations for the for the triphasic distribution of Archaea on human skin include exogenous factors such as the use of detergents, cosmetics, and soaps, affecting the surface microbiome in general.

Our study provides evidence that specific eury- and mainly thaumarchaeal taxa are associated with human skin, represented by a core archaeome of the human skin. In particular, the amount of archaeal 16S rRNA genes was significantly higher in human subjects older than 60 years and younger than 12 years compared to middle-aged human beings. These results, together with results obtained from spectroscopy imaging, allowed us to link lower sebum levels and thus reduced skin moisture with an increase of archaeal signatures. Our study emphasizes, that ongoing studies of the skin microbiome are largely biased towards Bacteria, due to methodical limitations, but also due to the selection of healthy, middle-aged persons for such analyses. Based on the knowledge on Thaumarchaeota available so far, it seems this group of microorganisms could have an impact on the skin by lowering the pH, and by removal of certain nitrogen compounds. Nevertheless, the clinical relevance of Thaumarchaeota remains unclear and awaits further studies with respect to the pathogenic or salutogenic potential of archaea found on human skin.

## Methods

### Sampling and sample handling

Human skin-wipe microbiome samples were taken and handled with approval by and in accordance with the Ethics Commission at the University of Regensburg and all experiments were performed in accordance with relevant guidelines and regulations. The Ethics Commission stated that no ethical concerns are raised by the methods applied and approved the following procedures. Written informed consent was obtained from all study participants, or their legal representatives. No metadata were derived except age and sex of the participant, and received samples were treated pseudo-anonymized. Human material was not subject of this study.

Microbiome samples from the entire front torso were taken using DNA-free sponges (Copan Innovation; polyurethane, 2 × 35 × 40 mm; moistened with 5 ml sterile isotonic saline solution (0.9% NaCl (w/v), DNA-free) by the volunteers themselves or their parents, after a detailed instruction by the research team members. The volunteers, who all lived in the same region of Germany, had not taken a shower right before sampling and have not applied any cosmetics that day. Each participant handed over the sample immediately after self-sampling, and the sponges were stored on ice after sampling and frozen (−80 °C) as soon as possible. An overview of all human skin wipe samples is shown in Supplementary Table S1. All samples (51 in total) were subjected to quantitative PCR, a subset thereof was processed for Archaea (21)- and Bacteria (2)-targeted next generation sequencing (NGS; Supplementary Table S1). FTIR-FPA hyperspectral imaging was performed on an additional set of samples (10 in total, Supplementary Table S1), as this method requires a specific sample preparation. These samples were taken from different volunteers and at different time points than the samples for sequencing-based analysis.

### Sample preparation for FTIR-FPA hyperspectral imaging

Microbial cells were detached from the sampling sponges with 0.9% (w/v) NaCl solution, and the suspension was handled according to the requirements of the methods of FTIR spectral analyses. Briefly, three milliliters of the cell suspension prepared from each subject were separated into two 1.5 ml fractions. Fraction one was subjected to DNA extraction and quantitative PCR as described^15^. Fraction two was fixed with formaldehyde (1 h at room temperature) and then washed twice (centrifugation) with 0.9% (w/v) NaCl solution. For the infrared FTIR hyperspectral imaging analysis, the formaldehyde-fixed cells were suspended in 0.9% (w/v) NaCl solution and shipped to the Berkeley Laboratory. As negative controls, extracts from sterile sampling sponges and, as a positive control, *Nitrososphaera viennensis* cells^20^, kindly provided by C. Schleper, were processed.

### DNA extraction from samples, quantitative PCR of bacterial and archaeal 16S rRNA gene sequences and subsequent statistical analysis

The procedure for DNA extraction from samples is given in the supplementary method information.

Quantitative PCR was performed with primer pairs 338bf/517ur (Bacteria) and 344af/517ur (Archaea), respectively, with a final primer concentration of 300 nM following the protocol described previously^15^. Positive controls for amplification and quantification were *Nitrosopumilus maritimus* and *Staphylococcus warneri*; as negative control, DNA extract from an untreated sponge was used, which was processed exactly like the samples. Copy numbers detected in skin-wipe samples were corrected by the mean of the run negative control (triplicate amplification of extraction blank), as the archaeal negative control revealed minor signals derived from primer-dimerization, as reported earlier^15^. For each sample, amplification was performed in triplicates and results were only used for further analyses when a) the efficiency of the run was above 90%, b) R2 values were above 0.99, and c) triplicate amplifications showed a standard deviation of less than 0.25.

Human subjects were grouped by age ( < 12, 12–60, > 60 years) due to different skin constitutions in these age ranges (see discussion). Percent abundance of Archaea in these groups was tested for normal distributions (Shapiro-Wilk test). Normal distribution of data was rejected as the Shapiro-Wilk test revealed highly significant p-values. Differences in abundance distribution of Archaea between age groups were tested using a Kruskal-Wallis test followed by a Dunn’s test to correct for multiple comparisons. False discovery correction was applied to p-values using the Benjamini-Hochberg metric. All tests were performed in the R programming environment^52^ with the aid of the dunn.test library.

### Amplification of archaeal 16S rRNA genes and next generation sequencing (NGS), processing of the reads and phylogenetic analysis

The procedures are explained in the supplementary method information. From all DNA extractions, covering the broad age-range of the volunteers, 21 samples (see Supplementary Table S1) were selected for NGS using 454 pyrotag-technology. Besides archaeal amplicons, bacterial amplicons were produced for two of the samples to verify that our skin microbiome analysis procedure worked properly, that the retrieved samples were representative of the human skin microbiome and to exclude biases in the sampling methodology (Supplementary Table S6). Datasets were submitted to NCIB Sequence Read Archive (SRA) and are publicly available (BioProject ID PRJNA313528).

### Fourier Transform Infrared (FTIR) focal plane array (FPA) hyperspectral imaging and analysis

Wafer chips with duplicates of 5-µl aliquots of skin-wipe microbial samples or controls were placed under a stream of sterile air. They were allowed to dry to form a thin layer of scattered microbial cells. A negative control (pure NaCl physiologic solution) and a positive control *Nitrososphaera viennensis* (approx. 10^7^ cells per ml) was processed. The detection/identification of Archaea in each binned pixel was based on our in-house database of known bacterial and archaeal standards^21, 22^ (see Supplementary Fig. 1 for details).

The detailed description is given in the supplementary method information.

## Electronic supplementary material

Supplementary information, including Supplementary Figures

Supplementary Table S 1

Supplementary Table S 2

Supplementary Table S 3

Supplementary Table S 4

Supplementary Table S 5

Supplementary Table S 6
