# Supplementary material for: Human age and skin physiology shape diversity and abundance of Archaea on skin
Source: Sci Rep. 2017 Jun 22;7:4039. doi: 10.1038/s41598-017-04197-4 (PMC5481324; doi:10.1038/s41598-017-04197-4)
Supplement: Supplementary file 1 — Supplementary information, including Supplementary Figures [file 41598_2017_4197_MOESM1_ESM.pdf]

Human age and skin physiology shape diversity and abundance of Archaea on skin
-Supplementary Figures-

Christine Moissl-Eichinger [1,2,*] , Alexander J. Probst[3], Giovanni Birarda[4,7], Anna Auerbach[5], Kaisa Koskinen[1,2], Peter Wolf[6], Hoi-Ying N. Holman[7]

[1] Medical University of Graz, Department for Internal Medicine, Auenbruggerplatz 15, 8036 Graz, Austria
[2] BioTechMed Graz, Krenngasse 37, 8010 Graz, Austria
[3] Department of Earth and Planetary Science, University of California, Berkeley, 307 McCone Hall, Berkeley, CA 94720, USA
[4] Elettra – Sincrotrone Trieste, Strada Statale 14 - km 163,5 in AREA Science Park, 34149 Basovizza, Trieste Italy
[5] University of Regensburg, Department for Microbiology and Archaea Center, Universitaetsstr. 31, 93053 Regensburg, Germany
[6] Medical University of Graz, Department for Dermatology, Auenbruggerplatz 8, 8036 Graz, Austria
[7] Berkeley Synchrotron Infrared Structural Biology Program, Lawrence Berkeley National Laboratory, One Cyclotron Road, Berkeley, California, United States of America
* Corresponding author.

Supplementary Figure S1: FTIR-FPA spectra of archaeal and bacterial reference strains. Based on their different membrane architecture, archaea can be easily distinguished.

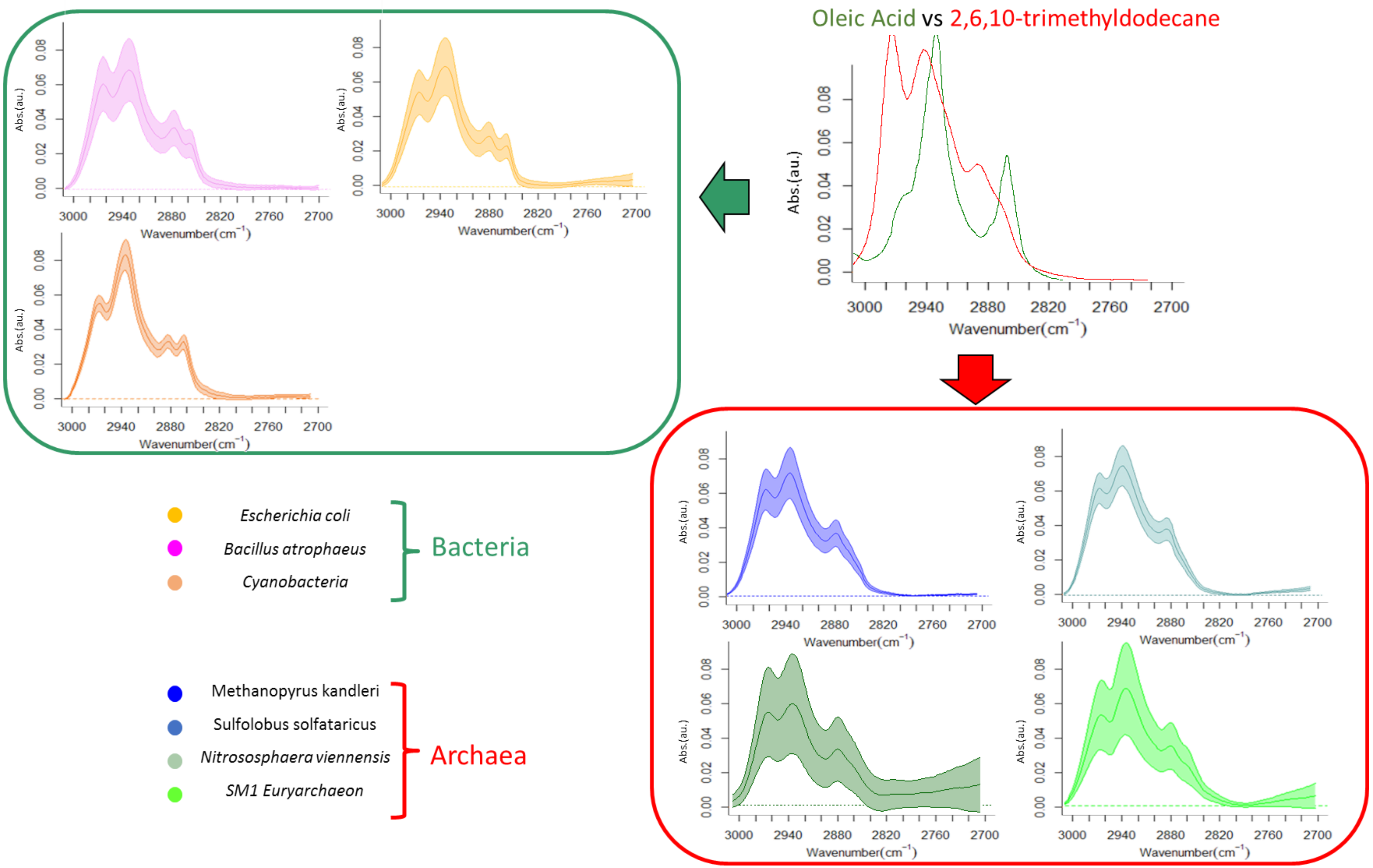

Supplementary Figure S2: Biplot of the Lipid/Protein ratio versus (A) the archaeal IR count and (B) the qPCR values indicates trends that support the hypothesis that people with drier skin may host more archaea cells.

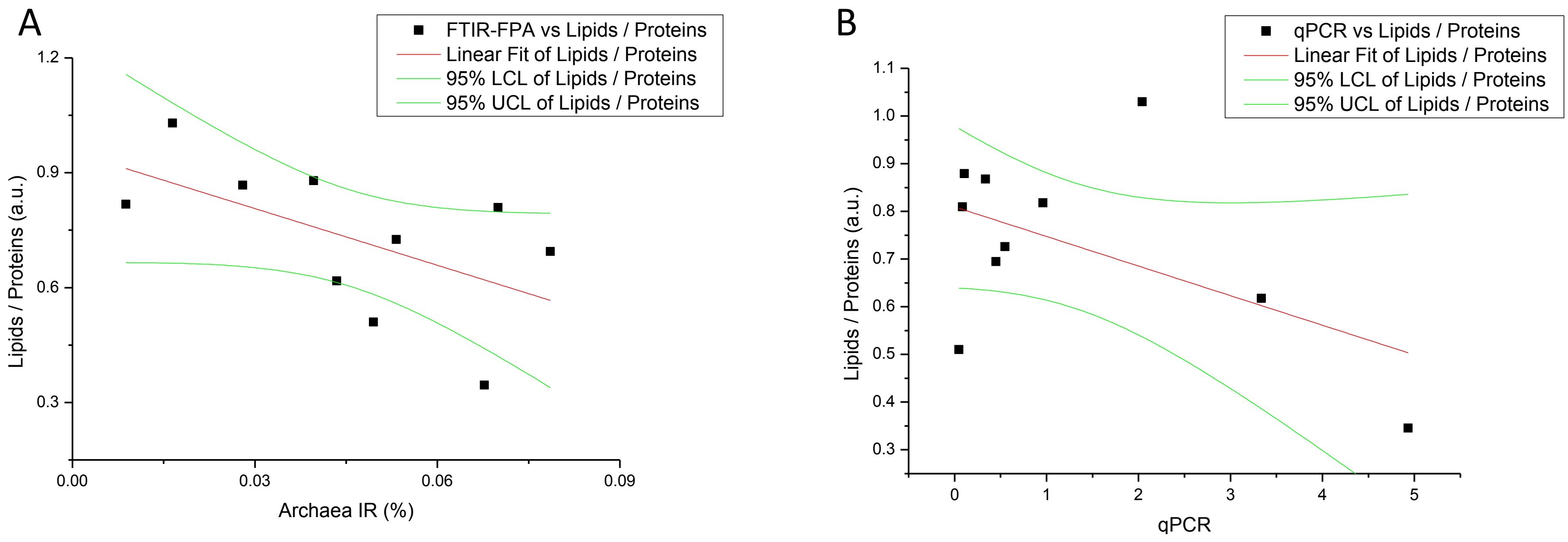

Supplementary Figure S3: Display of the human skin archaeomes of different skin wipe samples as revealed in this study. This chart is based on all archaeal sequences (reads) obtained via next generation sequencing of all 21 skin wipe samples. Sample 14.m.28 is not shown, since no archaeal reads were obtained. Sample names, given in the middle of each chart, refer to sample number, sex (w=female, m=male), and age (separated by "."). Thaumarchaeota are shown in red, Euryarchaeota in green, Crenarchaeota are indicated in blue.

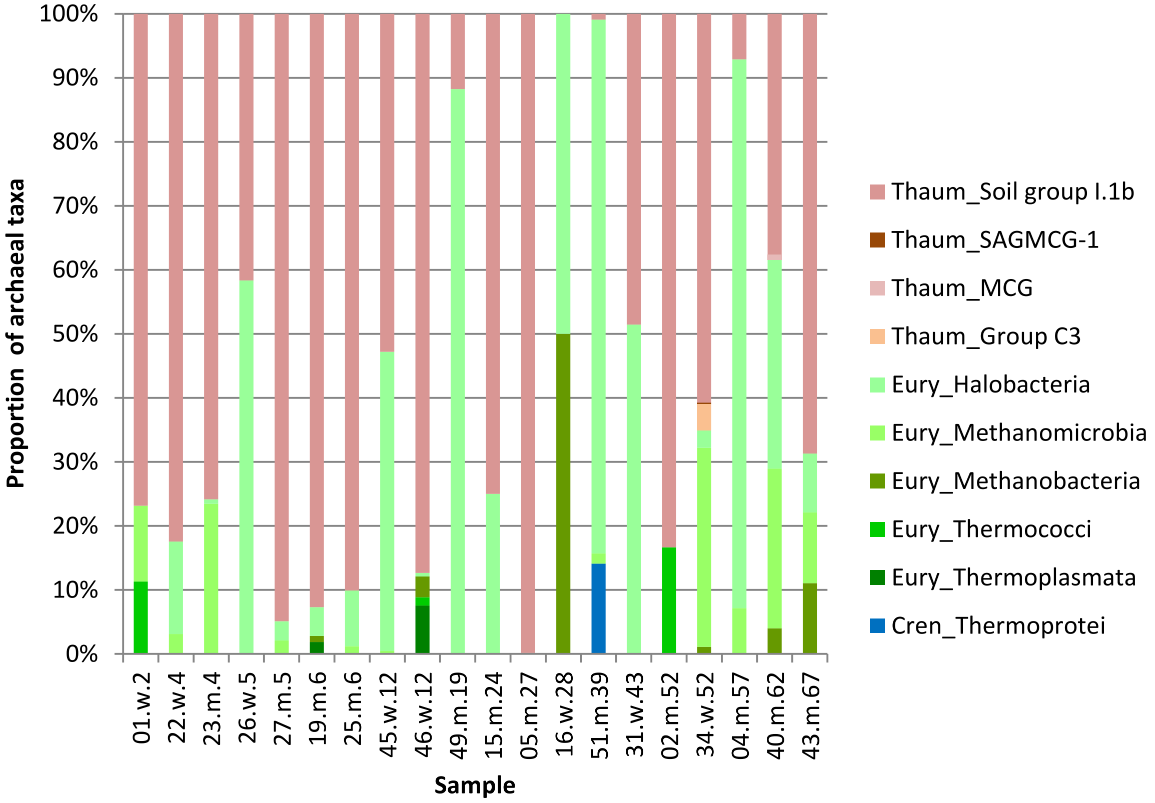

Supplementary Figure S4: Bubble plot of the 20 most abundant archaeal OTUs. Relative abundance is reflected by the size of the square (see legend). Samples are grouped according to their age group (age group I: 1-11 years, II: 12-60 years, III: >61 years), as indicated in the figure. Sample names, given in the middle of each chart, refer to sample number, sex (f=female, m=male), and age (separated by ".").

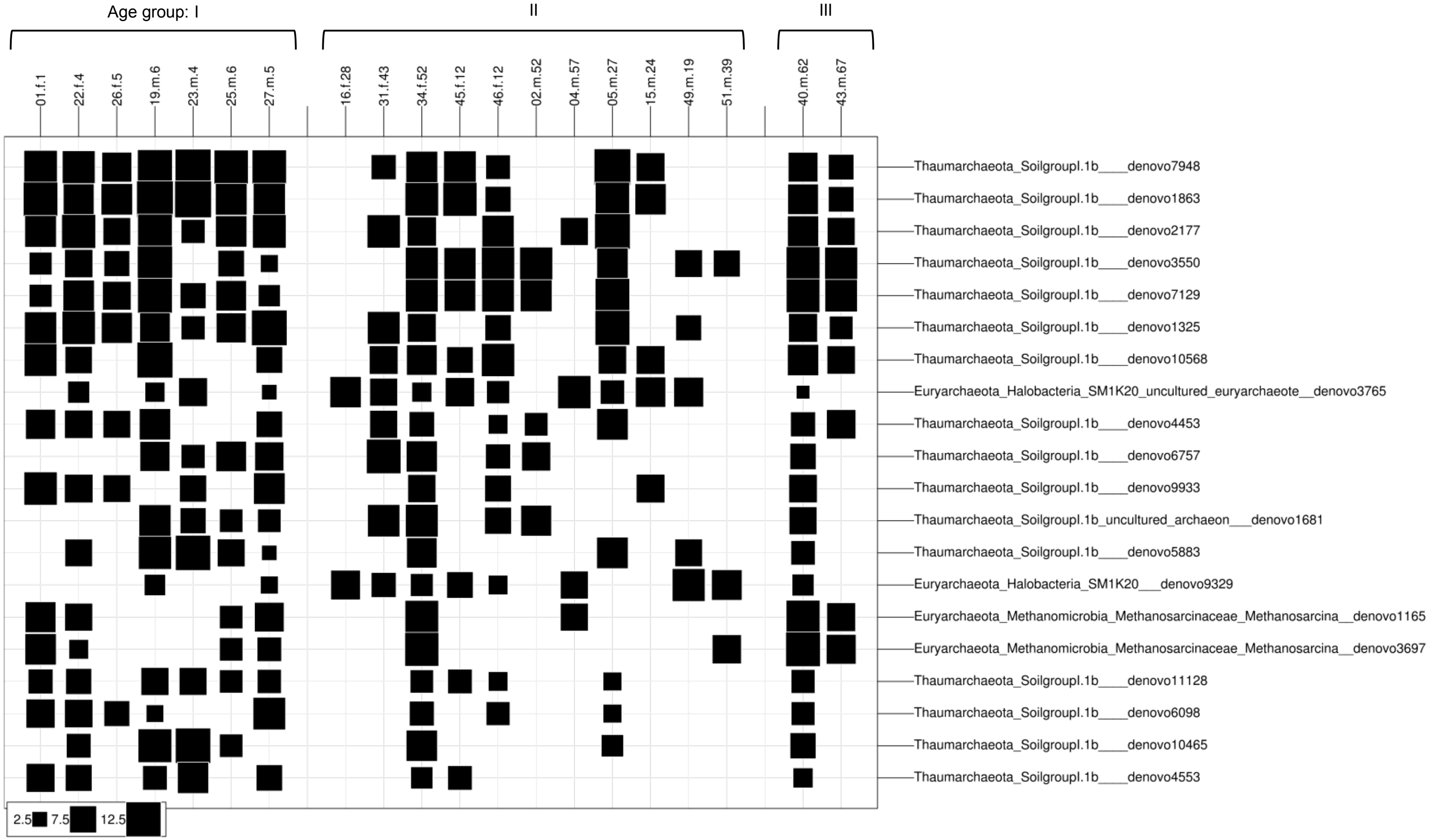

Supplementary Figure S5: Inverse Simpson Index, reflecting the diversity and richness in the archaeal skin communities. No significant differences were found with respect to age group (age group I: 1-11 years, II: 12-60 years, III: >61 years) and sex (f=female, m=male). However, a trend towards a lower index was found in age group II.

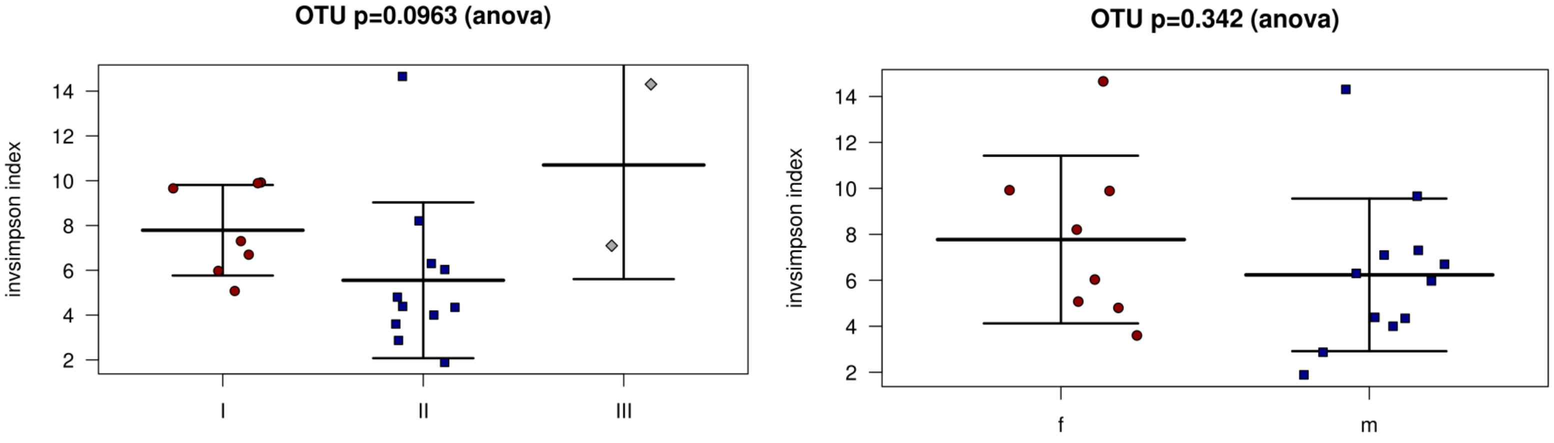

Supplementary Figure S6: Shannon Index, reflecting the diversity of the archaeal skin communities. No significant differences were found with respect to sex (f=female, m=male). However, significantly different diversities were retrieved for the age groups (age group I: 1-11 years, II: 12-60 years, III: >61 years) , with the lowest shannon index in age group II.

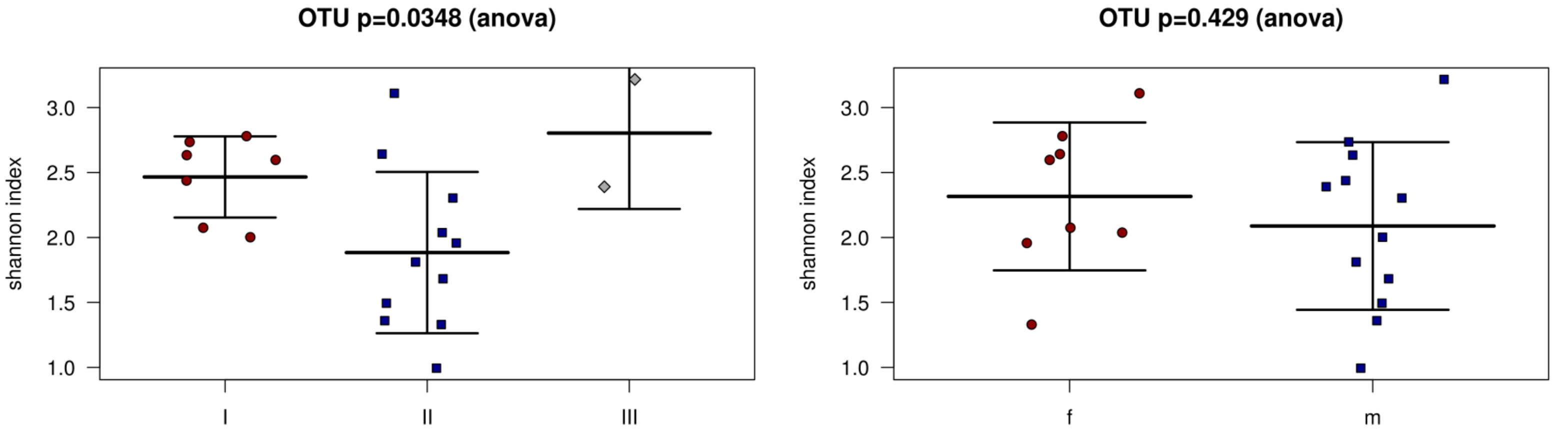

Supplementary Figure S7: Redundancy analysis plots on OTU level, indicating how well samples can be separated according to sex and age group. Age groups were found to be significantly associated with variation in the data matrix (p-values are given in the graphs).

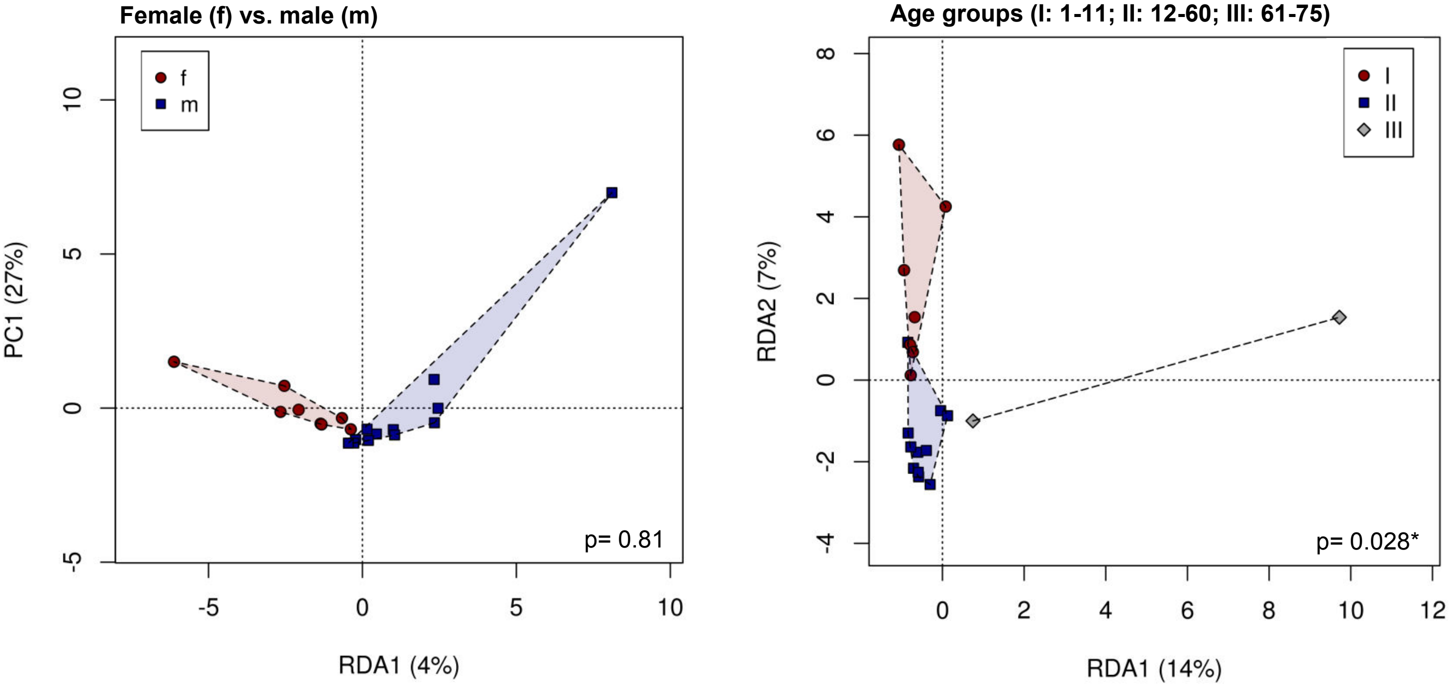

Supplementary Information: Methods

Human age and skin physiology shape diversity and abundance of Archaea on skin

Running title: Human skin archaeome

Christine Moissl-Eichinger [1,2,*], Alexander J. Probst[3], Giovanni Birarda[4,7], Anna Auerbach[5], Kaisa Koskinen[1,2], Peter Wolf[6], Hoi-Ying N. Holman[7]

[1] Medical University of Graz, Department for Internal Medicine, Auenbruggerplatz 15, 8036 Graz, Austria

[2] BioTechMed Graz, Krenngasse 37, 8010 Graz, Austria

[3] Department of Earth and Planetary Science, University of California, Berkeley, 307 McCone Hall, Berkeley, CA 94720, USA

[4] Elettra – Sincrotrone Trieste, Strada Statale 14 - km 163,5 in AREA Science Park, 34149 Basovizza, Trieste Italy

[5] University of Regensburg, Department for Microbiology and Archaea Center, Universitaetsstr. 31, 93053 Regensburg, Germany

[6] Medical University of Graz, Department for Dermatology, Auenbruggerplatz 8, 8036 Graz, Austria

[7] Berkeley Synchrotron Infrared Structural Biology Program, Lawrence Berkeley National Laboratory, One Cyclotron Road, Berkeley, California, United States of America

DNA extraction from samples

DNA was extracted following the protocol established previously [1].In brief, sponges were extracted for two minutes by sonication (Bandelin Sonorex Super 10 P); the sponges were squeezed and obtained liquid was removed for further extraction. This procedure was repeated after adding another 3 ml isotonic saline solution (0.9% NaCl (w/v), DNA-free). Both liquid fractions were pooled, and volume was adjusted to 3 ml. One aliquot of the liquid was subjected to XS-buffer extraction as described previously; the extraction procedure included a bead-beating step [1].

Amplification of archaeal 16S rRNA genes and next generation sequencing (NGS)

From all DNA extractions, covering the broad age-range of the volunteers, 21 samples (see Supplementary Table S1) were selected for NGS using 454 pyrotag-technology. Besides archaeal amplicons, bacterial amplicons were produced for two of the samples to verify that our skin microbiome analysis procedure worked properly, that the retrieved samples were representative of the human skin microbiome and to exclude biases in the sampling methodology (Supplementary Table S6).

Amplicons were produced using barcoded primer pairs 344af/915ar (Archaea, 5'-ACGGGGYGCAGCAGGCGCGA-3'/ 5'-GTGCTCCCCCGCCAATTCCT [2,3]) and 341f/805r (Bacteria, 5'-CCTACGGGNGGCWGCAG-3'/ 5'-GACTACHVGGGTATCTAATCC-3' [4]). For both runs, 35 PCR cycles were performed, with annealing temperature 60°C and 55°C, respectively. Amplicons were verified by gel electrophoresis, quantified via Qubit, and pooled for NGS sequencing (Roche 454 GS FLX). Library production and sequencing was performed by Macrogen Corporation (Geumcheon District, South Korea).

Processing of the reads, visualization and phylogenetic analysis

Raw reads (sff files) were processed using Qiime (version 1.9.0; [5]) following the standard operating procedures, which included chimera removal by usearch. Operational taxonomic units (OTUs) were

set at a 97% level (method: open reference picking, uclust [6]), taxonomical assignment was based pynast alignment [7] and on SILVA database 111 [8]. The small portion of bacterial 16S rRNA genes co-amplified in the Archaea-targeting approach (10-50%, depending on the sample) were filtered and removed from the dataset. In addition, OTUs represented with less than 5 reads in the dataset were removed. For visualization (RDA, bubble plot) and statistical analyses (inverse Simpson index and Shannon index), Calypso [9] was used. For these analyses, data were normalized using total-sum normalization (TSS).

For phylogenetic tree construction, a representative sequence from each OTU was used. This sequence data set was aligned using SILVA SINA [10] and then processed in MEGA6 [11]. Alignment was minimized and cropped to the core area, on which tree calculation (maximum likelihood) was based on. The obtained tree and the data were visualized using itol [12] and Krona [13].

Datasets were submitted to NCBI Sequence Read Archive (SRA) and are publicly available (BioProject ID PRJNA313528).

Fourier Transform Infrared (FTIR) focal plane array (FPA) hyperspectral imaging & analysis

Silicon double-side polished wafer chips with duplicates of 5-µl aliquots of skin-wipe microbial samples or controls were placed under a stream of HEPA filtered sterile air. They were allowed to dry to form a thin layer of scattered microbial cells on the wafer chip at room temperature. Two types of controls were processed: A negative control constituted of pure NaCl physiologic solution; a positive control constituted of *Nitrososphaera viennensis* (approx. 10^7 cells per ml) and two spiked samples with 25% and 75% of *N. viennensis* culture added to the liquid before being spotted on the wafer.

FTIR-FPA hyperspectral imaging measurements were performed at the infrared beamline 5.4 (Advanced Light Source, http://infrared.als.lbl.gov) on a Bruker GmbH System with a Hyperion 3000 IR VIS microscope coupled with a Vertex 70V interferometer and a mid-IR source (W546/8V globar). The microscope is equipped with a photovoltaic MCT (Mercury Cadmium Telluride) Focal Plane Array

(FPA) detector with 128x128 sensitive elements. The microscope is also equipped with a 36X and 0.5 N.A. Schwarzschild objective, granting a 145-by-145-μm^2 field of view with a 1.2-µm pixel size. All spectra were collected at the mid-infrared frequency range (4,000–1,000 cm^{-1} wavenumber) with a spectral resolution of 4 cm^{-1}. Each spectrum represented an average of 128 scans with a detection limit of approx. 250 0.5-µm cells (without pixel binning).

Spectra of the hyperspectral maps were pixel binned (with 2x2 pixel binning) to increase the signal-to-noise ratio. An approximately 450,000 binned spectra were analyzed and classified. Firstly, all binned spectra were subsequently corrected for the atmospheric contribution of water vapor and carbon dioxide, using a standard procedure implemented in OPUS 7.2 (Bruker GmbH). The recorded files were then converted in ENVI format and analyzed in R environment using the hyperSpec package [14] (http://hyperspec.r-forge.r-project.org). Total biomass in each skin microbiome sample was estimated in terms of (1) the absorption intensity of the protein amide I and amide II bands (1,700-1,480 cm^{-1}), and (2) of the lipids methyl ($-CH_3$) and methylene ($-CH_2$) groups (3,000-2,800 cm^{-1}) respectively.

The detection/identification of Archaea in each binned pixel was based on our in-house database of known bacterial and archaeal standards [15,16] (see Supplementary Fig. 1 for details). Bacterial membrane lipids consist of fatty acids with long alkylic ($-CH_2-$) chains that have only one to two terminal methyl ($-CH_3$) groups, whereas archaeal membrane lipids consist of branched and saturated hydrocarbon isoprene, and therefore relatively less $-CH_2-$ and more $-CH_3$ groups. For each pixel that had cells, we calculated the ratio of the infrared absorbance in the $-CH_3$ region (2,990–2,945 cm^{-1}) to that of the $-CH_2$ region (2,945–2,900 cm^{-1}). A threshold value of 0.75 was established to detect the presence of archaea in biofilm and environmental samples [1,16]. However, in this study of human skin microbiome, the threshold ratio was 0.65 according to the analysis of the spectra of the formaldehyde-fixed skin microbiome samples spiked with formaldehyde-fixed thaumarchaeal cells of different ratios.

Remark: The negative control shows a non-0 value due to the not avoidable instrumental error, and electrical noise. In some pixels, some electrical spikes can look as signals and be read as positive, so we can estimate this error with +/- 1% (Fig. 2).

References

1 Probst, A. J., Auerbach, A. K. & Moissl-Eichinger, C. Archaea on human skin. *PloS one* **8**, e65388 (2013).

2 Raskin, L., Stromley, J. M., Rittmann, B. E. & Stahl, D. A. Group-specific 16S rRNA hybridization probes to describe natural communities of methanogens. *Applied and Environmental Microbiology* **60**, 1232-1240 (1994).

3 Stahl, D. Development and application of nucleic acid probes. *Nucleic acid techniques in bacterial systematics* (1991).

4 Klindworth, A. *et al.* Evaluation of general 16S ribosomal RNA gene PCR primers for classical and next-generation sequencing-based diversity studies. *Nucleic acids research*, gks808 (2012).

5 Caporaso, J. G. *et al.* QIIME allows analysis of high-throughput community sequencing data. *Nature methods* **7**, 335-336 (2010).

6 Edgar, R. C. Search and clustering orders of magnitude faster than BLAST. *Bioinformatics* **26**, doi:10.1093/bioinformatics/btq461 (2010).

7 Caporaso, J. G. *et al.* PyNAST: a flexible tool for aligning sequences to a template alignment. *Bioinformatics* **26**, 266-267 (2010).

8 Quast, C. *et al.* The SILVA ribosomal RNA gene database project: improved data processing and web-based tools. *Nucleic acids research* **41**, D590-D596 (2013).

9 Zakrzewski, M. *et al.* Calypso: a user-friendly web-server for mining and visualizing microbiome–environment interactions. *Bioinformatics*, btw725 (2016).

10 Pruesse, E., Peplies, J. & Glöckner, F. O. SINA: accurate high-throughput multiple sequence alignment of ribosomal RNA genes. *Bioinformatics* **28**, 1823-1829 (2012).

11 Tamura, K., Stecher, G., Peterson, D., Filipski, A. & Kumar, S. MEGA6: molecular evolutionary genetics analysis version 6.0. *Molecular biology and evolution*, mst197 (2013).

12 Letunic, I. & Bork, P. Interactive Tree Of Life (iTOL): an online tool for phylogenetic tree display and annotation. *Bioinformatics* **23**, 127-128, doi:10.1093/bioinformatics/btl529 (2007).

13 Ondov, B. D., Bergman, N. H. & Phillippy, A. M. Interactive metagenomic visualization in a Web browser. *BMC bioinformatics* **12**, 1 (2011).

14 Beleites, C. & Sergo, V. hyperSpec: a package to handle hyperspectral data sets in R. *R package v. 0.98-20120725* (2012).

15 Probst, A. J. *et al.* Tackling the minority: sulfate-reducing bacteria in an archaea-dominated subsurface biofilm. *The ISME journal* **7**, 635-651 (2013).

16 Luef, B. *et al.* Diverse uncultivated ultra-small bacterial cells in groundwater. *Nature communications* **6** (2015).
